# Classification of voice disorder in children with cochlear implantation and hearing aid using multiple classifier fusion

**DOI:** 10.1186/1475-925X-10-3

**Published:** 2011-01-14

**Authors:** Zeinab Mahmoudi, Saeed Rahati, Mohammad Mahdi Ghasemi, Vahid Asadpour, Hamid Tayarani, Mohsen Rajati

**Affiliations:** 1 Islamic Azad University, Mashhad Branch, Department of Biomedical Engineering, Young Researchers Club, Iran; 2 Islamic Azad University, Mashhad Branch, Department of Electrical Engineering; 3 Mashhad University of Medical Sciences, ENT research Center, Department of Head and Neck Surgery, Iran; 4 Sajjad University of Mashhad, Department of Electrical Engineering, Iran; 5 Khorasan Cochlear Implant Center, Shenava-Gostar Institute, Iran

## Abstract

**Background:**

Speech production and speech phonetic features gradually improve in children by obtaining audio feedback after cochlear implantation or using hearing aids. The aim of this study was to develop and evaluate automated classification of voice disorder in children with cochlear implantation and hearing aids.

**Methods:**

We considered 4 disorder categories in children's voice using the following definitions:

Level_1: Children who produce spontaneous phonation and use words spontaneously and imitatively.

Level_2: Children, who produce spontaneous phonation, use words spontaneously and make short sentences imitatively.

Level_3: Children, who produce spontaneous phonations, use words and arbitrary sentences spontaneously.

Level_4: Normal children without any hearing loss background. Thirty Persian children participated in the study, including six children in each level from one to three and 12 children in level four. Voice samples of five isolated Persian words "mashin", "mar", "moosh", "gav" and "mouz" were analyzed. Four levels of the voice quality were considered, the higher the level the less significant the speech disorder. "Frame-based" and "word-based" features were extracted from voice signals. The frame-based features include intensity, fundamental frequency, formants, nasality and approximate entropy and word-based features include phase space features and wavelet coefficients. For frame-based features, hidden Markov models were used as classifiers and for word-based features, neural network was used.

**Results:**

After Classifiers fusion with three methods: Majority Voting Rule, Linear Combination and Stacked fusion, the best classification rates were obtained using frame-based and word-based features with MVR rule (level 1:100%, level 2: 93.75%, level 3: 100%, level 4: 94%).

**Conclusions:**

Result of this study may help speech pathologists follow up voice disorder recovery in children with cochlear implantation or hearing aid who are in the same age range.

## Background

Speech production strongly depends on hearing acuity. People who cannot adequately hear what they say, cannot correct errors in their speech production. Speech articulation in hearing impaired people under the age of 5 is disordered, of inadequate auditory feedback for speech sound acquisition [1,2]. Cochlear implantation (CI) or the use of hearing aids (HA) can partially or fully restore hearing. Consequently speech production can improve over time and enters the normal range. After hearing is restored, hearing impaired individuals use auditory feedback to adjust voice features such as voice intensity, intonation and vowel duration.

There are characteristics impairments of voice and speech disorders in deaf or newly rehabilitated hearing impaired people [1,3]. Speech resonance may be hyper nasal. The tongue may be carried in the back of the mouth which may cause some resonance problems. Phoneme production may be better at the beginning of the words. Poor monitoring of speech production results in deficits in the fundamental frequency, intensity and duration of voice. Consequently, production of high frequency vowels is more difficult. Furthermore, impaired fundamental frequency may be exacerbated by laryngeal muscle tension. These characteristic deficits reduce comprehension of deaf speech by strangers to no more than 20% to 25%. On the other hand when a child communicates with others verbally, the content is effective only 7% in conveying feeling; while, facial movements and voice are effective 55% and 38% respectively [4]. So analyzing voice plays an important part in evaluation of child's oral communications.

Few studies have categorized and classified the existing disorder of voice in impaired hearing children. Judgment about voice quality has been mainly subjective and depends on the listeners' skills such as SIR (Speech Intelligibility Criteria) [3]. Although, there are numerable reports that consider the influence of the hearing loss on the voice phonetic features quantitatively and objectively; none have fused this quantitative information to classify voice using quantified levels. Thus, creating an automatic system that can determine the state of the child's phonetic disorder and classify it as a specific level based on phonetic features may be essential to help speech pathologists evaluate and monitor voice recovery in children with hearing impairment. If the severity of voice disorder is not determined accurately, it may result in inadequate training and possible failure in speech recovery process after CI or HA. In this study, the methods that can classify speech disorder based on signal processing features are evaluated.

Various methods have been used by researchers to trace the effects of the different disorder and abnormalities on speech signals. In [1], improvement in acoustic features of the speech was studied in pre-lingually deaf children and in adults with hearing background. Both groups were fitted with CI. They were studied in 3 month intervals after implantation and compared with a control group for 15 months. A new criterion was used for measuring voice nasality in individuals with CI. Results showed that CI in patients enables them to make gradual improvement in segmental and supra - segmental features of speech including formants, fundamental frequency and nasality and after 15 months post-implantation, the difference between their voice parameters and those of the normal control group was at minimum. In another study [2], 20 pre-lingually deaf children were studied. Vowel /a/ was extracted from all recorded words and changes in fundamental frequency (f0) and 3 first formants (f1, f2, f3) were evaluated and compared with a control group. These studies showed that after CI, f0 decreased; formants did not increase or decrease in a specific trend but they became close to normal after some months post operation.

Changes in production of 3 main vowels were evaluated in another study [5]. Voice samples of 13 pre-lingually deaf children and 12 post-lingually deaf adults were analyzed in production of isolated vowels /a/, /u/ and /i/ before implantation and 6 and 12 months post implantation. The area of the vowel triangle was used to evaluate changes in acoustic features of vowels pre and post CI. The results showed that vowel triangle area is a sensitive indicator of the changes in vowel production after CI. In another study [6], 31 pre-lingually deaf children expressed isolated vowel / a / pre operation and 6, 12, 24 months post CI. Unlike the previous studies, this research did not report postoperative decrease in f_0_. The results of this study [6] indicated that CI enables children to control fundamental frequency and loudness of voice. Two Japanese cochlear implanted children participated in a study by [4]. Their voices were recorded monthly with first and second formants extracted for comparison with their mothers' formants. It was reported that their f1-f2 triangle was highly similar to their mothers' after one year post implantation. A similar study was done on children who used hearing aids [4]. Results identified that 12 months after the first experiment, the children's formants became close to their mothers', however the similarity between mother's formants and child's formants was much higher in children with cochlear implantation than children with hearing aid.

A further study of speech quality of impaired hearing children was classified according to the listeners' judgment using SIR criteria [7]. Using these criteria a person is categorized in one of the five qualitative levels based on their speech intelligibility. Further speech quality evaluation was completed in [8]. Jitter and shimmer of voice and also correlation dimension of speech attractors were extracted from 51 vowel samples of normal subjects and 67 vowel samples of subjects with paralyzed vocal cords. Results showed that all 3 mentioned features in patients had higher values than healthy subjects. In addition, classification results with support vector machine indicated that correlation dimension plays a more important rule than classic acoustic features in separation of patients from healthy subjects. In [9], four cochlear implanted children and four children with normal hearing as control group were included. A paragraph from a standard French text was read by children. Samples were evaluated using the subjective voice parameters of loudness, pitch perturbation, speech fluency and appropriate stops during speech production. Additionally, objective parameters including fundamental frequency, formants frequencies and vowels duration were extracted from the voice samples. Results indicated that sound intensity was different between the control group and cochlear implanted children. Also formants frequencies in implanted children were different from those in the control group, but this difference was not easily distinguishable. Subjective test did not show a significant difference between normal and implanted children but it was possible to establish a correlation between subjective and objective tests to evaluate implanted children's voice disorder.

The aim of this study was not to individually classify every identified characteristic voice impairment in individuals with hearing impairment, but to combine features of impairment to classify voice abnormality as a whole. Specifically, methods that can classify speech disorder based on signal processing features were evaluated. We aimed to combine the outcome data from these analyses to form an index for abnormality.

## Methods

### Proposed disorder levels in children's voice

For purposes of this experiment, we considered a scale consisting of four disorder categories to classify voice:

• Level 1: Children who produce spontaneous phonations and use words spontaneously and imitatively.

• Level 2: Children who produce spontaneous phonations, use words spontaneously and make short sentences imitatively.

• Level 3: Children who produce spontaneous phonations, use words and arbitrary sentences spontaneously.

• Level 4: Normal children without any hearing loss background.

The levels of voice disorder in this study were defined by the speech therapist. Since we proposed to develop a system that would correspond to existing subjective criteria, we limited classification of impairment to four levels, however the number of levels (voice categories) are expandable and can be increased if the needs arise. So, the resolution and accuracy of this quantitative estimate can be improved.

The purpose of this study is to categorize above levels and quantify them based on segmental features of children's voice. After CI or using HA, speaking skills of the children develop so that they use more words and sentences; phonation features also gradually improve. As a result, it is reasonable that at any stage of progress, their assigned severity level may change sufficiently to be distinguishable from the previous or next stage. In classification of the above levels, a modification of SIR criterion is used [10,11]. The criterion scores and their correspondence to our defined disorder levels are introduced in table 1.

**Table 1:** Comparison of defined disorder levels in the study with SIR criteria

SIR score	Levels of intelligibility in SIR criteria	Levels of voice disorder in the study
1	Connected speech is unintelligible. Prerecognizable words in spoken language, primary mode of communication may be manual.	Level1
2	Connected speech is unintelligible. Intelligible speech is developing in single words when context and lip-reading cues are available.	Level2
3	Connected speech is intelligible to a listener who concentrates on lip-reading.	Level2
4	Connected speech is intelligible to a listener who has little experience of a deaf person's speech.	Level3
5	Connected speech is intelligible to all listeners.	Level4

In total, thirty children between the ages of 3-6 years participated in the study. This included 18 children using CI or HA that were selected according to the speech therapist's subjective ranking from level 1 to level 3 and 12 normal children in level 4. Table 2 shows the demographic data related to the children in levels one to four.

**Table 2:** Demographic data of children participating in the study

Level of voice disorder	Average age (in month)	Average Age at CI or using HA (in month)	Average last time after CI or using HA (in month)	number of kids in each level
Level 1	52	45.3	18	6 (4 male & 2 female)
Level 2	58	38	20.16	6(4 male & 2 female)
Level 3	59	28.5	29.3	6(3 male & 3 female)
Level 4	72	-	-	12(8 male & 4 female)

### Recording speech

Voice samples of the 5 following isolated Persian words were recorded and analyzed for this study.

1. mashin/ mα: ∫in/

2. mar/ mα:r/

3. moosh/mu: ∫/

4. gav/gα: v/

5. mouz /moυz

The English translations are: 'car', 'snake', 'mouse', 'cow' and 'banana' respectively. These words were used as they contained 3 main Persian vowels: /i:/ in 'mashin', /α:/ in 'mar', /u:/ in 'moosh' and 3 Persian voiced consonants: /g/ and /v/ in 'gav' and /z/ in 'mouz'. Selection criteria for these words were that they were easily spoken by all children and they could be displayed in pictures to children. To avoid any imitative speech, each word was displayed via a picture in Microsoft Power Point slide show with 4 second intervals. The child was asked to tell the name of each word after seeing its picture. Each picture was repeated 7 times for every child to express. We recorded speech samples from 18 children with CI or HA (six children in each level) and 12 normal children. Sampling frequency was 44100 Hz. Voice samples were then analyzed and voice features were extracted.

### Extracting features from speech

The features used in this research are listed below:

### Relative Intensity (RI)

Intensity is an indicator of sound loudness. It has been shown that people with impaired hearing tend to speak louder than non-impaired people [3]. In this study, relative intensity of the voice, defined as the ratio of the intensity to the maximum intensity was extracted from each word.

### Formants

Transfer function of the human vocal tract from larynx to mouth is an all pole model that is expressed by Auto Regressive (AR) models [4]. Formants are poles of this transfer function and appear as peaks in the voice spectrum. They are different for each vowel and consonant. It is suggested that not any of the formants can independently explain a specific trend in voice recovery process after CI, but ratio of formants-for example f1/f2 in vowels and consonants- is a better indicator of progress path. It is speculated that at any stage of speech improvement after implant surgery, this ratio can identify the difference between the implanted and normal children's voice [5].

### Fundamental frequency (f_0_)

Fundamental frequency (f_0_) is the frequency with which the vocal cords fluctuate. f0 instability can be a sign of abnormality in the speech production system such as in cochlear implanted people[12]. It is reported that fundamental frequency in hearing impaired children is higher than normal children [1,3] and [13].

### Nasality

A common problem in producing speech by impaired audio-verbal children is hyper nasality [1].

The main reason for this problem is the inability to control movements of the soft palate that separates the nasal and oral cavities, thus switching between nasal phonation to vocal phonation. When producing nasal phonation, air flow exits through nose at the end of the vocal tract and when producing oral phonation, air flow exits through the mouth. Reduction in the first formant amplitude and appearing an extra peak near 1 kHz with a valley in the range of 700 - 800 Hz in the frequency spectrum, show severe nasal cavity opening and hyper-nasality of voicing as a result [1]. Some researchers propose that the difference between the first formant amplitude and the extra peak amplitude near 1 kHz is a reliable criterion to determine the degree of nasality [1]. We show this difference with:

(1) $$\frac{1}{Nasality}\propto {\text{Amplitude}}_{{\text{f}}_{1}}-{\text{Amplitude}}_{1\text{kHz}}$$

Amplitude in the above equation is measured in dB scale. It should be noted that when ${\text{Amplitude}}_{{\text{f}}_{1}}-{\text{Amplitude}}_{1\text{kHz}}$ increases, nasality decreases.

### Fractal dimension

Calculating phase space dimension of the signals is one of the most common strategies to estimate the degree of chaotic behaviour in these signals. This feature is based on measuring the localization of the trajectory points in the signal attractor while the system is exploring the time [14]. There are different algorithms to calculate dimension of the phase space including fractal dimension and correlation dimension. We used the Higuchi method to calculate fractal dimension of the voice signals. For more information on this algorithm see [15].

### Approximate Entropy (ApEn)

Approximate entropy, like fractal dimension measures the level of complexity and chaos in signals. Advantageous of ApEn over sample entropy and fractal dimension is that in calculating fractal dimension or sample entropy, a large number of data samples is required to have reasonable accuracy. However, for ApEn analysis, a much smaller data set is enough. See [16] and [17] for ApEN calculation.

### lyapanov Exponent

Lyapanov exponent is used to quantify chaos in the system and its symbol is λ [18].

If *^λ > 0^*, trajectory points escape from each other exponentially, the signal is chaotic.

If *^λ < 0^*, trajectory points get close to each other, the signal is deterministic.

If *^λ = 0^*, trajectory points remain at a fixed distance from each other, the signal is aperiodic.

### Energy of wavelet coefficients

The wavelet functions are reproductions of time shifting, stretching and folding a mother wavelet called ψ:

(2) $$\psi_{\text{j,k}}\left(\text{t}\right)={\text{a}}_{0}{}^{-\frac{\text{j}}{2}}\psi \left({\text{a}}^{-\text{j}}\text{t}-{\text{kb}}_{\text{0}}\right)$$

Wavelet coefficients of function fw(t) are:

(3) $${\text{d}}_{\text{j,k}}=<{\text{f}}_{\text{w}}\left(\text{t}\right),\psi_{\text{j,k}}\left(\text{t}\right)\geq (\frac{1}{a_{0}^{\frac{\text{j}}{2}}})\int {\text{f}}_{\text{w}}\left(\text{t}\right)\psi \left({\text{a}}_{0}^{-\text{j}}\text{t}-{\text{kb}}_{\text{0}}\right)\text{dt}$$

In the current study, the energy of the wavelet coefficients was extracted from speech signal that is the mean square of the wavelet coefficients. The mother wavelet used in the study was an order five Gaussian function. We extracted wavelet coefficients in the scales of seven, eight and nine.

Wavelet transform provides good accuracy in both time and frequency domains. So it is a suitable tool to analyze non-stationary signals such as speech. This property makes it theoretically appropriate to evaluate speech after CI [19].

Table 3 summarizes the introduced features extracted from voice signal. Fundamental frequency and formants were extracted using Autocorrelation Coefficients in Praat software. Other processes were performed in Matlab software. In all processing, 25 ms Hamming windows with 75% overlap are used.

**Table 3:** Input features to the recognition system

features	description
f0	Fundamental frequency of the voice signal
RI(Relative Intensity)	Ratio of intensity to the maximum intensity in syllable.
f1	Frequency of the first formant
f2	Frequency of the second formant
f3	Frequency of the third formant
f1/f2	Ratio of first to second formant frequencies
Nasality (1/(Af1-A1k))	Reverse of the difference between amplitude of the first formant and spectral extra peak at 1 kHz
Entropy	Approximate entropy of the voice signal
Fractal dimension	Fractal dimension of the speech phase space
Lyapanov exponent	Lyapanov exponent of the voice signal
Mean energy of Wavelet coefficients	Mean energy of Wavelet coefficients in scales 7,8 and 9

### Hidden Markov Models (HMM) as classifier

HMM consists of limited number of hidden states that connect to some observable states via probabilities. Every hidden state depends only on the N previous states [20] and [21]. In order to use HMM classifier, a feature vector comes out from every hidden state of the system. In fact feature vector is the sequence of the observable states in the model.

To train HMM models, Expectation Maximization algorithm (EM) is used [21]. HMM Training is the estimation of the transition probabilities from every hidden state to another hidden state or another observable state. Details about HMM structure and its algorithms are given in [21]. To use continuous structure of HMM (mHMM), observation vector is modelled by some mixture Gaussians using k-means algorithm. This new observation vector is given to HMM model and using EM, transition probabilities are estimated repeatedly and finally optimized model is achieved recursively. In order to classify by HMM, the number of the trained HMMs should be the same as the number of the classes. For example if there are k classes of patterns, there should be k trained HMMs.

Then log-likelihood of the given observation sequence for all k HMMs is calculated using Forward algorithm. Observed state sequence belongs to the i-th HMM and so to the i-th voice category if this model maximizes the log-likelihood of the observed sequence. The HMM structure used in this study to represent each word is Left-Right structure with 8 hidden states. Hidden Markov Models are continues type with four Mixture Gaussians for each state.

### Multiple classifier fusion

**Figure 1:**
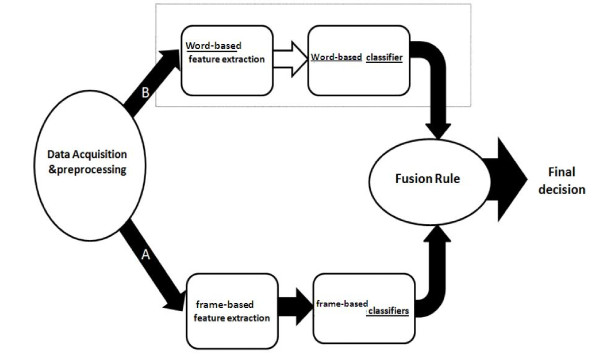
**Main diagram of the study**.

Information fusion can be used at different stages of the processing [22]; data fusion, feature fusion or classifier fusion. In the current study classifier fusion was considered. Every word acted as an isolated classifier for classifying four levels (classes) of voice disorder and then final decision was made by fusing classifiers in a hierarchical arrangement [23]. In the base of this structure, an input decision was made about each word and then at higher stage, these decisions were combined [19]. Two different sets of features were extracted from recorded voice signals including "frame-based" and "word-based" features. Frame-based features are: fundamental frequency, the first, second and third formants, first to the second formant ratio, relative intensity of the voice, nasality and approximate entropy. Word-based features were: fractal dimension, Lyapanov exponent and mean energy of the wavelet coefficients. For the frame-based features, hidden Markov models were used as classifiers and for the word-based features, neural network was used. Classifier outputs including word-based and frame-based were then fused together in a hierarchical scheme. Figure 1 shows the main diagram of the study.

For the HMM classifiers, first the log-likelihoods of the given sequence of independent observations (extracted features from test data) were calculated for the given word by using Forward algorithm. Then the classifier chose the voice category which the HMM of that category indicated the highest log-likelihood among other HMMs. After testing all of the classifiers for all five isolated words, we had five highest log-likelihoods each of which related to one of the words "/ mα: ∫in/, / mα:r/, /mu: ∫/, /gα: v/ and /moυz". In fact, we had five decisions (e.g. each word gave out one decision about the voice category of the child); however, we needed just one decision about the voice category that the child was situated in. Thus, we had to fuse these five decisions into one decision. So, using classifier fusion was mandatory. We chose the fast, easy and reliable methods of classifier fusion for our purpose. Since we needed fusion rules that could be used online with sufficient accuracy, we could not use complicated and time consuming methods. We decided to use these three methods of fusion: Majority Voting Rule, Linear Combination and Stacked fusion and compare them with each other.

**Figure 2:**
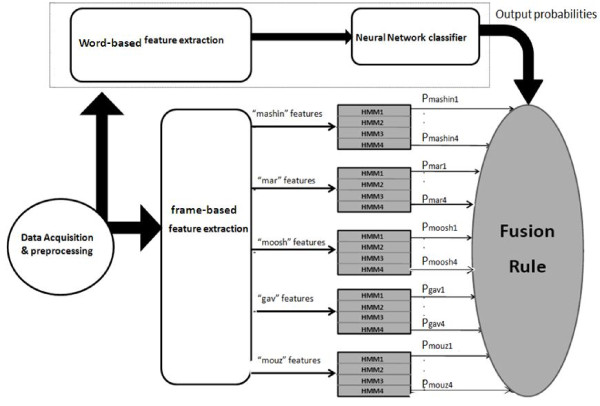
**Detailed diagram of the system**.

None of the words could independently classify all levels of the disorder, so a fusion of the words is essential to make a reliable decision on the degree of disorder of the child's voice. For each level in each word, a Hidden Markov Model is trained, thus in total 20 HMMs (four HMMs for each word) were considered. Log-likelihoods of all HMMs were then fused to make a final decision. Figure 2 shows the detailed diagram of the study. In this structure, Markov models of each word are placed in parallel with HMMs from other words. Three methods of classifier fusion used in the study are described below. The purpose of all fusion rules is making a final decision based on the output information from all classifiers.

### Linear combination fusion

Linear combination is a fast and easy method to fuse classifiers. In this method, classifiers probabilities are simple or weighted averaged as in formula (4). 'x' is the feature-vector sequence. ${\text{p}}_{\text{i}}^{\text{k}}$(x) is the log-likelihood of the given feature-vector sequence x when this sequence is input of the i-th HMM of the k-th Classifier in this study referred to each word from the list "/ mα:∫in/, /mα:r/, /mu:∫/, /gα:v/ and /moυz". There should be a trained HMM for each voice category (voice level). Every word contained the four voice categories and there were five words. So, we had twenty HMMs totally (four HMMs for each word) which every four HMMs made an isolated classifier. Thus, we had N classifiers that N = 5 here (number of words) and we have 4 HMMs that i = 1...4 (i-th HMM corresponds to the i-th voice category). Further explanations can be found in [22].

(4) $$p_{\text{i}}^{\text{ave}}\left(\text{x}\right)=\sum_{\text{k}=1}^{\text{N}}w_{k}p_{i}^{k}\left(x\right)$$

When the voice category of a child is going to be determined, he or she should utter all the five words mentioned in the paper. Then the extracted feature vector of each word was given to every of the four HMMs of that word and then four log-likelihoods were calculated. When done for all words, this combined to twenty log-likelihoods, each four of them belong to one classifier. To reduce this large number of likelihoods to ease the classification, we summed the log-likelihoods of the HMMs of the same categories from all the classifiers to finally reach to four log-likelihoods. Then the child belonged to the category which the sum of its HMM likelihoods is maximum.

We used simple averaging for fusion. Simple average is the optimal fuser for classifiers with the same accuracy and the same pair-wise correlations. Weighted average is required for imbalance classifiers, that is, classifiers with different accuracy and/or different pair-wise correlations [22].

### Majority Voting Rule (MVR) fusion

Let us consider the N abstract ("crisp") classifiers outputs S(1), ..., S(N) associated to the pattern x. Class label c_i _is assigned to the pattern x if c_i _is the most frequent label in the crisp classifier outputs [22]. To implement this method in the current study, first every word was classified in one of the 4 disorder classes (labels), and then the final voice label was the most frequent label assigned by all 5 words.

### Stacked fusion

In this method, the k soft outputs of the N individual classifiers can be considered as features of a new classification problem. In other words, classifiers can be regarded as the feature extractors. Another classifier can be used as fuser: this is the so-called "stacked" approach [22]. We used a multi-layer perceptron (MLP) neural network as the final classifier with one hidden layer, 6 neurons in the input, 10 neurons in the hidden and 4 neurons in the output layers. Training algorithm is back propagation. Transfer function of the hidden neurons is tangent sigmoid and for output neurons it is log sigmoid.

### Data division in classifiers

**Figure 3:**
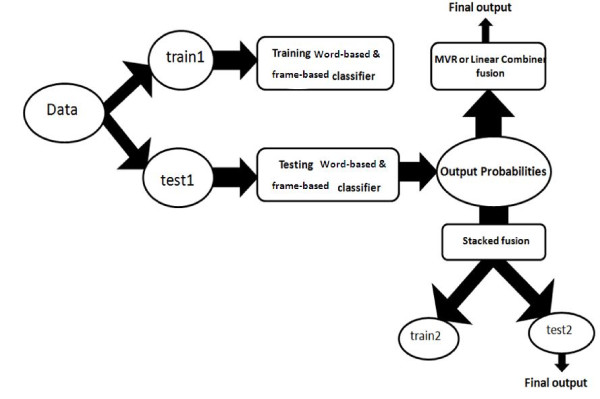
**Data division in different parts of the system**.

Figure 3 illustrates division of the train and test data in different parts of the system. Data were first divided to two main parts including train1 and test 1. Train1 was used to train frame-based and word-based classifier. Test1 was used to test them. Train1 incorporated 60% of the whole data and test1 contained 40% of the data. Then the log-likelihoods of classifiers were divided depending on the fusion rule. If MVR or Linear Combination is used, there is no extra division in data and final test data would be achieved from the whole test1, but in the case of using stack fusion, log-likelihoods are divided to two separated parts including train2 and test2 to train and test final classifier, so test data of the whole system would be achieved from test2 that is a fraction of test1.

The data was divided such that the recordings from a random batch of all children are used for training and the trained model is used to classify other data. Train data is independent from test data. This means that the data samples were never present in both train and test set at the same time but occasionally and randomly there may be some children who are present in both train and test data. Since division of train and test was done on a random permutation of the total data, there may be some children who were in both train and test data set; however their voice samples are not common in train and test. Although some data samples of train and test may be from the same children, they are different utterances of those children.

The question may arise if 'this system can be generalized to other groups of children, the answer is yes. The authors set a test to examine this. The classification accuracies were checked in two situations: in the first situation among the total data there were some children, who were present in both train and test, and in the second situation there was no common child in test and train data. The classifiers are were and tested several times for each situation and the results of classification were averaged out. There was no significant difference between classification accuracies of the two situations. This provides evidence that the system can be generalized.

After recording voice samples, frame-based and word-based features were extracted from the signal. Fundamental frequency, intensity and formants were extracted using Praat software. Other signal processing was performed in Matlab software. We used method in [1] to quantify nasality in this study. In frame-based feature extraction, processing is done with 25 ms hamming windows with 75% overlap.

## Results

Table 4 represents correct classification rates of the specified levels for each word before any classifier fusion. These percentages were achieved using Random Sub Sampling cross-validation [24].

**Table 4:** Classification rate for all words using frame-based features^a^

word	Level 1 accuracy	Level 2 accuracy	Level 3 accuracy	Level 4 accuracy
'mashin'	86.87	80	98.12	66.7
'mar'	91.25%	71.25%	100%	51.51%
'moosh'	83.12%	67.5%	100%	86.97%
'gav'	90%	40%	100%	94%
'mouz'	87.5%	60.62%	100	95.76%

^a ^Features: f_0_, f_1_, f_2_, f_1_/f_2 _, f_3_, RI, nasality and approximate entropy

As it was expected and the results show as well, every word was classified well at just some levels and not all of them. For example, the word "mar" was acceptably classified in levels 1 and 3 but classified poorly at levels 2 and 4 (low classification rate), so a combination of all 5 words was required to make a reliable decision about the disorder level of the child who has spoken all the 5 words.

**Figure 4:**
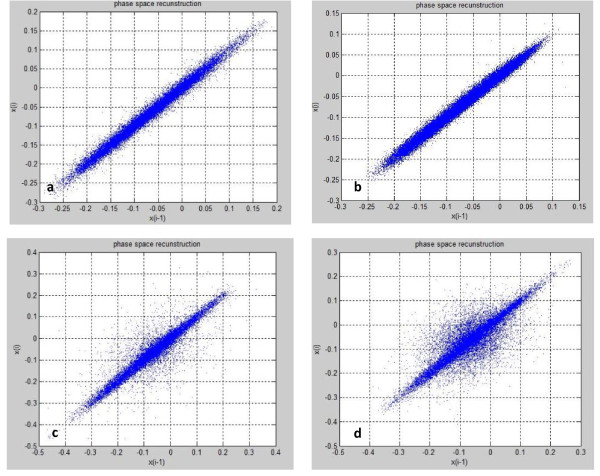
**Phase space reconstruction of the voice samples from the word 'mouz'**. a: Phase space of 'mouz' signal from level 1. b: Phase space of 'mouz' signal from level 2. c: Phase space of 'mouz' signal from level 3. d: Phase space of 'mouz' signal from level 4.

Figures 4a, b, c and 4d show the reconstructed phase space of the voice samples from the word 'mouz'. x(t) and x(t-1) on the figures axes are samples of the raw speech signal.

It can be seen that when severity of the disorder level decreased, phase space extends and stretches in dimension.

From the phase space of a signal, with a fast interpretation, we found the degree of chaos existing in the signal. The more chaotic a signal, the more stretched its phase space. A more chaotic signal was produced from a system with more flexible behaviour and higher dynamic dimension. Therefore it was concluded that in the children with milder voice disorder, the speech production system has greater ability to produce flexible phonation and the child can match suitable segmental features to different parts of a word, so phase space is more self organized and more chaotic. However the children with more severe voice disorder cannot manage acoustic features in different parts of their speech and their voice was raw and unsophisticated. Table 5 shows final classification rates for different subgroups of features by 3 types of classifiers fusion. Percentages less than 50% were determined with 'not classified'. The train set and test set were independent and separated. So it was an out-of-sample classification and the results were predictive accuracies. All the percentages mentioned in the tables, including tables 4 and 5, are the results of classification on test data set which is independent from train data set. To measure the accuracies in table 5 Random Sub Sampling cross-validation was used as it was used to measure the accuracies in table 4. In this type of cross-validation the train and test data division was repeated randomly for several times and the results were achieved by averaging the classification accuracies over several randomly testing the classifiers.

**Table 5:** Average classification rate for subgroups of features and different fusion rules

subgroup	Features	Fusion method	Level 1 accuracy	Level 2 accuracy	Level 3 accuracy	Level 4 accuracy	Average accuracy in all levels
1	f0, f1, f2, f1/f2, f3, RI, nasality, approximate entropy	Stacked fusion	not classified	54.0%	75.0%	80.0%	65.7%
		MVR	93.8%	68.8%	100.0%	87.9%	87.4%
		Linear combination	93.8%	87.5%	100.0%	93.9%%	93.8%

2	f0, f1, f2, f1/f2, f3, nasality, approximate entropy, fractal dimension	Stacked fusion	63.0%	65.2%	76.3%	82.0%%	71.2%
		MVR	100.0%	81.2%	100.0%	91.0%	93.1%
		Linear combination	87.5%	81.2%	100.0%	100.0%	92.2%

3	f0, f1, f2, f1/f2, f3, RI, nasality approxiamate entropy, lyapanov exponent	Stacked fusion	not classified	not classified	71.2%	79.4%	62.5%
		MVR	100.0%	75.0%	100.0%	91.0%	91.5%
		Linear combination	87.5%	81.2%	100.0%	94.0%	90.7%

4	f0, f1, f2, f1/f2, f3, RI, nasality approxiamate entropy, fractal dimension, lyapanov exponent, wavelet coefficients in 3 scales	Stacked fusion	60.0%	62.0%	80.0%	87.5%	73.8%
		MVR	100.0%	93.8%	100.0%	94.0%	96.9%
		Linear combination	100.0%	68.8%	100.0%	100.0%	92.2%

In this study, 40% of total data was used as test and the rest 60% was used as training data set. Training and testing the classifiers was repeated ten times. In each repetition, test and train data were divisions of random permutations of total data and this permutation was repeated randomly each time. The percentages mentioned in the tables were averaged accuracies over ten times randomly training and testing the classifiers. This method was used in all classifiers including frame-based, word-based and stacked fusion classifiers.

The best results and the highest classification rate for each level were achieved from features "fundamental frequency, first, second and third formants, the first to the second formant ratio, relative intensity, nasality, fractal dimension, lyapanov exponent and energy of the wavelet coefficients" when we used MVR fusion rule (level1:100%, level2:93.75%, level3: 100% and level4: 94%).

## Discussion

In this study, voice disorder in children with cochlear implantation and hearing aids are classified quantitatively and objectively. These children gradually make improvement in their speech production after gaining audio-feedback by using cochlear implantation or hearing aid. We considered 4 levels of disorder in the children's voice. The four levels of the voice disorder were defined mainly with regard to the higher-level linguistic capabilities of the child (for example "use words spontaneously and make short sentences") that are usually correlated with the lower-level phonetic articulation aspects of the child's speech (nasality, formant frequencies, fundamental frequency, etc). However, there might be cases with language disorder that cannot be detected by an instrument that only measures articulation aspects of speech.

Linear and nonlinear features including: "fundamental frequency, first, second and third formants, the first to the second formant ratio, relative intensity, nasality, fractal dimension, lyapanov exponent and energy of the wavelet coefficient" were extracted from the voice in expressing five Persian words and were classified in a hierarchical structure. In the first level of this structure, there were HMMs and a neural network. Outputs of all the classifiers were then fused by three methods.

Considering the information in table 2, we can assume that the longer the period of CI or HA usage, the better the speech will be. This is what others have also previously mentioned [1-3,6,9,25] and [26]. In table 2 we have also shown that the children who are implanted at earlier ages, attain higher levels of speech with fewer abnormalities in their voice, which is reported by Eberhard as well [5]. It can be seen in table 2 that older children have higher levels of speech than younger children. Older children have greater ability to control phonetic features of their voice due to more sophisticated speech system. This is matched with the result of [9].

In another study [7] speech quality of impaired hearing children was classified according to listeners' judgment using Speech Intelligibility Rating (SIR) criteria. In the mentioned study, no quantitative and objective classification is applied to the children's voice; while, in the current study voice disorder were categorized quantitatively based on speech processing features.

Considering the diversity of children at each level, results of this study can be used to help speech pathologists follow up voice disorder recovery in children with the same range of age that use cochlear implantation or hearing aid. This system can be an effective strategy to evaluate methods to train these children. By doing this detection test, speech therapists will understand whether the applied training was effective or not and whether the child is in its appropriate level regarding the type and the duration of the training.

Different educational strategies are implemented to rehabilitate hearing impaired children; however, direct comparison of these strategies without taking a proper quantitative criterion will not be possible. Quantifying voice disorder in these children and expressing it in the form of a level, give speech pathologists the chance to compare different training strategies and choose the best one. In addition, designing a website with the engine system created in this study, in order to provide special facilities for patients undergoing the speech therapy, gives this opportunity to these patients to connect to this website according to a scheduled time table and upload samples of their voice to the site to be analyzed by the system. Then the analyzer motor implemented in the site will be able to process the speech sample, including convenience feature extraction, classification and quantification in order to perform an approximately online diagnostic test. Finally the result of the test and state of the voice improvement can be released or can be sent via monthly or daily email to the patient.

As with most classification studies, in which a new objective technique is being tested against a human expert (e.g., an experienced speech therapist), the ever-present 'gold standard' problem becomes an important issue. In this paper, the system is being trained and tested against a single human expert who will also be prone to making errors. Thus, the classification errors made by the system may not have been errors at all and may have reflected some noise in the human expert which a panel of other human experts may have disagreed with. Thus, the gold standard, particularly a single one, is unlikely to be perfect in classification of such a complex process such as speech.

## Conclusions

In this study for the first time, nonlinear and phase space features are extracted from voice of the children with cochlear implantation and hearing aid. Results showed the capability of nonlinear analysis to follow up speech recovery in these children. This result is also achieved in analyzing speech disorder in patients with laryngeal abnormality to separate healthy subjects from patients [14].

Comparing Table 5 with Table 4 it is observed that after combining outputs of the classifiers, the final classification rates in all levels increase compared with isolated classifiers. To have better classification accuracy in fused classifiers than isolated classifiers, two conditions must be met: 1-Isolated classifiers should have high accuracy. 2- Output errors of the isolated classifiers should be independent [27]. In other words, classifiers should complement and compensate for each other, otherwise, regardless of what the fusion rule is, results of the multiple classifier fusion would not be better, and perhaps even worse, than isolated classifiers. Considering table 4, it can be seen that output errors of classification by each word almost does not overlap other words. Levels that cannot be classified well by a word are classified well by at least one other word. Therefore after fusing classifiers, better results are achieved compared to isolated classification. Best classification accuracy is gained from features of "fundamental frequency, first, second and third formants, the first to the second formant ratio, relative intensity, nasality, fractal dimension, lyapanov exponent and mean energy of the wavelet coefficient" with MVR fusion rule.

Linear Combination is a fast and easy method for fusion when a large number of classifiers are to be fused. This method has been one of the most successful and common ways for fusion of multiple classifiers [28]. Stacked fusion made improvement in classification rate compared to individual classifiers but the amount of increase in accuracy is not as MVR and Linear Combination. When using stacked fusion, the meta-classifier should be trained with a data set different from the one used for the individual classifiers (Experts' boasting Issue) [22]. Data is therefore divided to 3 smaller subsets; first to train the individual classifiers, second to train the meta-classifier and third to test the whole system. When the amount of data is small, a subdivision of it would not be enough to train meta-classifier, so the MLP network used as meta-classifier in this study cannot be trained well and the results of the classification are not acceptable.

## Competing interests

The authors declare that they have no competing interests.

## Authors' contributions

ZM carried out data acquisition and processing and drafted the manuscript. SR supervised the study as the first supervisor. MMG co supervised the study and prepared the suitable condition to record the children's voice at his educational institute (Shenava Gostar Institute). VA helped ZM in signal processing studies. HT as a speech therapist labelled the children's voice disorder and prepared the suitable condition for recording the children's voice at his educational institute (Shenava Gostar Institute). MR corrected the drafted manuscript.
